# Building a sustainable future for eLife

**DOI:** 10.7554/eLife.21230

**Published:** 2016-09-29

**Authors:** Randy Schekman, Mark Patterson

**Keywords:** scientific publishing, open access, peer review, eLife, publishing

## Abstract

To support the long-term growth of eLife we are going to introduce a publication fee of $2500.

The first research articles in eLife were published in 2012. Since then, thanks to the support of our three funders, thousands of authors have benefited from eLife’s editorial and publishing processes at no cost to themselves. We have been able to maintain this approach for four years, and it has no doubt contributed to making eLife an attractive place to publish high-quality science. However, given the continuing increase in submissions we are experiencing – on track to exceed 8000 manuscripts this year – we have reached the point where we need a revenue stream to help cover the costs of future growth. We will therefore introduce a publication fee of $2500 for all research papers submitted on or after January 1, 2017 that go on to be accepted for publication.

Our editorial policy is to publish all the articles that meet the high standards set by our editors.

We believe the time is right to introduce this fee, to help build our editorial board and to support our growing community of readers, reviewers and authors. Together with the renewed support of our funders, which we announced earlier this year, the revenue from publication fees will ensure that eLife continues to flourish.

We have calculated the fee so that it will cover the marginal cost that is incurred for every additional article we publish: payments to editors is the largest component of these costs. The remaining, fixed costs (which include staff, technology and marketing costs) will be covered by our funding. Consequently, as the journal continues to grow, the increase in our publishing costs will be met by the publication fee income. This will enable us to scale our publishing operation with confidence, and will mean that many more scientists will be able to benefit from eLife’s editorial process as authors, reviewers and readers.

Our editorial policy is to publish all the articles that meet the high standards set by our editors. Unlike journals whose publishing decisions are based at least in part on the expense of the printed page, as an online journal eLife has no quota or target number of pages. Our biggest challenge, therefore, will be to continue to recruit active scientists to our editorial board so that we can maintain the quality and integrity of the eLife editorial process.

The peer review process at eLife involves much consultation amongst our editors and it works because these editors are committed to improving the way science is evaluated and presented. Many eLife editors have had experience as editors for other journals, sometimes paid, sometimes not. At eLife, given the time and energy that is being expended by our editors, we feel it is important to compensate them at least partially for their work, and payments are offered to the Editor-in-Chief, Deputy Editors, Senior Editors and Reviewing Editors (who orchestrate our consultative peer review process). In addition to these editorial costs, there are marginal costs associated with administration of the editorial process, with the operation of the publishing infrastructure, and with the processing and distribution of the accepted manuscript files. (More details on our costs are available in this blogpost.)

We had also considered a submission fee to cover some of the costs of the editorial process. eLife is a selective journal, so a great deal of effort often goes into submissions that are ultimately not judged to meet the standards set by the editors. A submission fee would have helped to cover the costs of this part of the process, but given the variation in the feedback that is given to authors, we felt that a publication fee would be fairer.

Our editors are committed to improving the way science is evaluated and presented.

At the same time, we must ensure that our publication fee is not a barrier to any author who lacks sufficient funds to cover the fee, and that it provides good value for money. Our research suggests that a fee of $2500 is reasonable relative to the fees charged by other journals and given that funds for open-access fees are increasingly being made available by institutions and funders. However, we appreciate that there will often be circumstances when authors do not have access to sufficient funds, and in these cases the fee will be waived.

In terms of value for money, there are many benefits that authors gain from publishing in eLife. First and foremost is the clear and constructive feedback delivered by our editors. Authors also value the space to present their findings in full, along with all associated figures, datasets and media files. Early-career researchers in particular benefit from our efforts to increase the exposure and reach of their work by, for example, media outreach and support for travel to conferences. Together with the benefit of being able to make their work immediately and openly available to readers throughout the world, we hope that eLife authors will consider the eLife publication fee to be good value for money.

In addition to supporting the growth of eLife as a publisher, publication fees will allow us to use more of the support provided by our funders for R&D into new products and services. eLife has already produced two publishing tools that are openly available to all: the eLife Lens reader provides a new interface for reading and navigating complex research articles; and eLife Continuum is a technology platform for continuous open-access publishing. Several other projects are currently underway, and we will continue to invest strongly in such work so that the potential for improvements in the digital communication of new research can start to be realised.

Over the past few months we have shared financial information about eLife in an effort to encourage similar transparency by other publishers and journals. There are many different approaches to publishing, each associated with different costs and benefits. There has been limited sharing of cost information, historically, making it difficult to have meaningful discussions about the value of different approaches. We need data to inform how we build a more effective and efficient system for research communication for the long term.

We are steadily moving publishing into the digital era, but have yet to take full advantage of the possibilities on offer. Indeed many practices still reflect the legacy of print publishing. We hope that eLife can continue to shed these legacy practices, and that we can convince many more scientists, particularly those who are in the early stages of their careers, to entrust their very best work to eLife and to join us in our efforts to make publishing work better for science.

